# Influences of Biochar on Bioremediation/Phytoremediation Potential of Metal-Contaminated Soils

**DOI:** 10.3389/fmicb.2022.929730

**Published:** 2022-06-09

**Authors:** Mathiyazhagan Narayanan, Ying Ma

**Affiliations:** ^1^Department of Biotechnology, Division of Research and Innovation, Saveetha School of Engineering, Saveetha Institute of Medical and Technical Science, Chennai, India; ^2^College of Resources and Environment, Southwest University, Chongqing, China

**Keywords:** heavy metals, biochar, plants, bioremediation, phytoremediation

## Abstract

A number of anthropogenic and weathering activities accumulate heavy metals in soils, causing adverse effects on soil characteristics, microbial activity (diversity), agricultural practices, and underground aquifers. Controlling soil heavy metal pollution is difficult due to its persistence in soils, resulting in the deposition and transmission into the food web *via* agricultural food products, ultimately affecting human health. This review critically explores the potential for remediation of metal-contaminated soils using a biochar-based responsible approach. Plant-based biochar is an auspicious bio-based residue substance that can be used for metal-polluted soil remediation and soil improvement as a sustainable approach. Plants with rapid growth and increased biomass can meet the requirements for phytoremediation in large quantities. Recent research indicates significant progress in understanding the mechanisms of metal accumulation and contaminant movement in plants used for phytoremediation of metal-contaminated soil. Excessive contamination reduces plant biomass and growth, which has substantial hyperaccumulating possibilities and is detrimental to the phytoremediation process. Biochar derived from various plant sources can promote the growth and phytoremediation competence of native or wild plants grown in metal-polluted soil. Carbon-enriched biochar encourages native microbial growth by neutralizing pH and providing nutritional support. Thus, this review critically discusses the influence of plant and agricultural waste-based biochar on plant phytoremediation potential in metal-contaminated soils.

## Introduction

Land degradation and soil contamination are a persistent threat to humans’ and the environment’s wellbeing (Azam, 2016). Heavy metal and metalloid intensification in soil have increased rapidly in terms of natural phenomena and anthropogenic activities, including mining, agricultural activities, and industrial and municipal discharge, which all pose severe threats to environmental protection and public health (Sharma et al., 2022). Because they are non-biodegradable, they might remain in the soil, enter into the food chain *via* agricultural crops, and even accumulate in humans *via* biomagnification/bioaccumulation (Gogoi et al., 2021). Heavy metals are a class of elements distinguished by their high atomic weight and mass, with a specific density of greater than 5 g/cm^3^ (Buha et al., 2014). There are 21 non-metals, 16 light metals, and 53 heavy metals among the 90 naturally occurring elements (Gholizadeh and Hu, 2021). Such elements are divided into two categories: those that could be needed in trace amounts (Cu, Zn, Ni, Fe, V, Mn, Co, and Mo) by certain organisms and others such as Pb, As, Cd, and Hg, which are entirely considered as dangerous (Naja and Volesky, 2017). Heavy metals in their natural state are not available for root uptake or are not accessible to living beings. Anthropogenic sources, such as battery manufacturing, mineral extraction (mining), explosives, pesticides, herbicides, chemical fertilizers, and effluent irrigation, cause an excessive increase in such elements, contributing to their deposition and distribution (Dutta and Sharma, 2019). When these activities exceed acceptable levels, they endanger all living beings and have disastrous effects on their concentration. These elements are tolerant in various ways depending on the life form with which they are confronted (Villa-Achupallas et al., 2018). Metal pollution has posed a significant risk to human health as well as the environment due to its toxic nature. Hence, remediation of metal pollution from soils is critical (Rathour et al., 2022). Numerous remediation strategies depending on their mobilization or immobilization mechanisms have indeed been established to address these issues (Akcil et al., 2015; Wang et al., 2021). However, they are typically very expensive, and planned remediation is often delayed due to the absence of sufficient funds (Yrjälä and Lopez-Echartea, 2021). Unique nature-based substances are emerging that ought to be cost-effective in remedial work but necessitate further development because they require useful insights into the structure–function relationships (Byrne et al., 2018). Biochar, a carbon-rich component, is thought to play an important role in the bioavailability of heavy metal-polluted soil, resulting in biotransformation and bioremediation (Yu et al., 2019). However, biochar is frequently produced from various feedstocks using different pyrolysis processes; hence, the surface characteristics may vary significantly (Yu et al., 2019). Plants and biochar blending can be used to enhance the *in situ* or on-site bioremediation. Nevertheless, this is critical to address a few essential lines of study to ensure the safe and long-term use of biochar. Biochar is being developed for use in the environmental cleanup of both inorganic and organic contaminants, and their integration with phytoremediation is an excellent option (Rodriguez-Franco and Page-Dumroese, 2021). Since then, biochar-blended phytoremediation has grown in popularity as a groundbreaking technology for enhancing the phytoremediation potency in metal-polluted soils (Muthusaravanan et al., 2020). Various biochar properties demonstrate their influence on heavy metal transport, mobilization, and precipitation, improving soil structure, the release of nutrients, and microbial diversity, thus supporting plant growth (Yuan et al., 2019). This review presents the environmental influence and applications of biochar-blended phytoremediation of heavy metal-polluted soils and their interaction with plants during remediation.

## Agricultural Wastes for Biochar Fabrication

**FIGURE 1:**
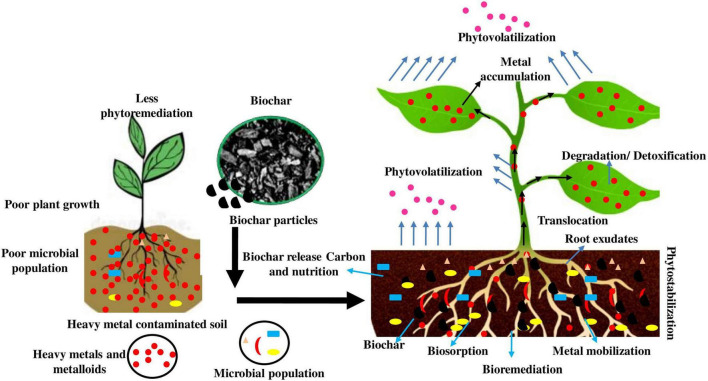
Biochar fabrication from various agricultural wastes.

The primary ingredients for biochar production are agricultural, forestry, household, and livestock waste (Figure 1), which are all abundant across the world. Agricultural waste has previously been used in a limited number of applications, including as a renewable source and animal feed (Spiertz and Ewert, 2009). Another report stated that the nationwide possibilities for producing biochar from agricultural biomass have been calculated and predicted to be around 3.1 million tons of biochar from around 10.7 million tons of biomass (Awasthi et al., 2021). The highest biomass is derived primarily from rice husk, which has a yield of 6.8 million t y^–1^ and can produce biochar up to 1.77 million t y^–1^, accounting for approximately 56.48% of the total nationwide biochar fabrication potential (Susilawati et al., 2020).

## Biochar Properties

### Physicochemical Properties

Biochar is being used as a soil conditioner and is acquired at low temperature pyrolysis—ranging from 400 to 700°C—of numerous biomasses including manure (cow dung), agriculture waste (wastes of maize, sugarcane, weeds, and so on), and biosolids in the absence of oxygen. It is thus differentiated from charcoal (Khiari et al., 2019). The physical and chemical properties of biochar obtained from wood, agricultural residues, poultry manure, or sludge at various pyrolysis temperatures are summarized in Table 1. Although the physicochemical characteristics of biochar diversified substantially due to the fabrication from a wide range of feedstocks using varying pyrolysis processes, biochar is usually basic in nature with such a large specific surface area, huge porosity, changeable charges, and different functional groups, as mentioned in Table 1. Such properties can also have an impact on pH, conductivity (CEC), and surface adsorption capacities. Biochar particle size is determined by the standard particle size of the feedstock; nevertheless, it is usually much smaller due to shrinking and attrition during the pyrolysis process. Due to the improved tensile strength of raw materials at higher pyrolysis temperatures, it may yield smaller-sized biochar particles (Albalasmeh et al., 2020). The functional groups on the surface of biochar, porous structure, and ionic charges can aid in the physical adsorption (Zhang et al., 2020), co-precipitation (Chen et al., 2022), complexation, mobilization/immobilization (Hu et al., 2020), and detoxification (Alrashidi et al., 2020) of metal pollutants and support the hyperaccumulator’s phytoremediation potential.

**TABLE 1:** Physical and chemical properties of biochar obtained from various plant residues and manure.

Biochar materials	pH	Temperature: °C (pyrolysis)	CEC (mmol kg^–1^)	Carbon (%)	Carbon/ Nitrogen ratio	Total phosphate (mg kg^–1^)	Elements (%)						Surface area (m^2^ g^–1^)	Volatiles (%)	Ash (%)	References
							
							Ca	Fe	Mg	N	P	K				
Rice husk	8.9	300–400	37.3	23.4	–	–	0.21	0.26	0.18	0.73	0.48	0.54	–	–	44.35	Susilawati et al., 2020
Oak wood	3.7−6.4	60−600	75.7−182	47.1−87.5	444−489	5−29							450−642	27.5−88.6	0.3−1.3	Lehmann et al., 2011; Sun et al., 2018
Palm bunches	9.39	350−450	9.9	42.33	−		0.4	0.5	0.67	0.99	0.49	8.65	−	−	27.09	Susilawati et al., 2020
Pine needles	6.4−10.6	300−700	−	84.2−93.7	22−26	−	−	−	−	−	−	−	4.1−391	6.2−38.6	7.2−18.7	Sun et al., 2018
Bamboo	9.30	350−450	9.30	50	−	−	0.16	0.16	0.13	1	0.45	3.18	−	−	11.26	Sun et al., 2018; Susilawati et al., 2020
Corn stover	6.7−9.4	60−600	252−459	42.6−70.6	51−83	526−2,114	−	−	−	−	−	−	293−527	23.5−85.2	8.8−16.7	Susilawati et al., 2020
Coconut shell	9.61	250−350	9.61	29.69	−	−	0.29	0.29	4.43	1.28	0.52	2.96	−	−	48.96	Sun et al., 2018
Chicken litters	8.2−10.3	60−700	58.7−363	7.9−38	10−25	493−16,685	−	−	−	−	−	−	1−94	18.3−60.5	16.9−72.5	Susilawati et al., 2020
Sludge	4.9−12	400−700	−	20−20.4	8.4−17	528−740	−	−	−	−	−	−	−	15.8−25.7	63.3−72.5	Sun et al., 2018
Palm cake	8.30	350−500	8.30	23.73	−	−	0.09	0.04	0.30	0.87	0.44	0.72	−	−	59.32	Susilawati et al., 2020
Branch legume	9.4	−	7.05	18.11	−	−	−	−	−	0.58	0.1	1.11	−	−	−	Sun et al., 2018
																

### Nutritional Property

Biochar consists of a variety of nutrients, including K, Mg, K, Ca, and P, which are derived from the pyrolysis raw material. During pyrolysis, the dissolved organic material is also formed (de Figueiredo et al., 2021). Hence, the biochar amalgamation could provide plants and microorganisms with bioavailable nutrients. The quantity and the type of the bioavailable nutrient content in biochar, on the other hand, are highly dependent on the raw material (feedstock) and pyrolysis conditions (Yang et al., 2019).

The elements such as C and N in biochar differed significantly while obtained from pine trees, poultry manure, and peanut husk at 400 and 500°C, respectively (El-Bassi et al., 2021). Furthermore, transferable phosphate, potassium, calcium, and magnesium were significantly higher in biochar produced at 500°C than in biochar produced at 400°C (Ferjani et al., 2019). The deviation was primarily associated with the high pyrolysis temperature, which enhanced raw material mineralization besides reduced CEC. From this standpoint, obtaining nutrient-enriched biochar from a nutrient-enriched raw material under appropriate pyrolysis conditions is critical (Ma et al., 2015). In fact, plant-derived biochar appear to have a reduced nutrient composition than biochar derived from manure (Embrandiri et al., 2012).

### Constancy Property

When biochar is applied to the soil, it appears as a separate particulate matter that differs from many other kinds of solid organic materials, which are either encapsulated in soil pore spaces or adsorbed on the mineral surfaces and obscured in aggregate particles (Kumar et al., 2018). Biochar with much more aromatic black carbons on the exterior seems to be more consistent in soil than other forms of organic carbons, thus improving carbon storage potential in soil properties (Lian and Xing, 2017). A previous study reported that the biochar mineralization rates are very low, with carbon half-lives up to 100 years (Williams et al., 2019). According to another investigation, perfect biochar particles were found in soils in wet tropical climates including the Amazon for millennia (Agegnehu et al., 2017).

## Biochar – Metal(Loid) Interaction

Biogeochemical interactions in the ecosystem have a significant impact on the destiny, transfer, and conversion or modifications of metals and metalloids (Breda et al., 2018). Because ionic metals and metalloids can occur in both anionic and cationic aspects, their behavior will be influenced by interactions with anionic and cationic charges of the biochar surface (Fijałkowska et al., 2021). When combined with topsoil, biochar with negative charges can strongly adsorb positive components (e.g., Cd^2+^ and Pb^2+^), whereas biochar with cationic charges can maintain anionic metal(loid)s (e.g., arsenite and arsenate) (Gupta et al., 2021). Adsorption mechanism, surface (co)precipitation, and surface complexation with active functional moieties are the major mechanisms for the immobilization of cationic metals (including Pb^2+^) and metalloids through biochar (Gupta et al., 2021). Thus, the biochar-stimulated improvements in soils, including increased soil pH, can reduce the bioavailability of cationic metals and metalloids even further. Since the physical and chemical attributes of biochar depend on the raw material type and pyrolysis circumstances (e.g., temperature and frequency of temperature rise), it is essential to recognize appropriate raw materials for biochar fabrication that have the efficiency to remediate various metals and metalloids in specific soils (Akhil et al., 2021). Anionic metalloids, including Cr, Se, and As, are frequently found as dominant species in soils with alkaline pH compared to cationic metalloids that are poorly adsorbed by negatively charged soil (Gupta et al., 2021).

The redox potential of metals and metalloids can influence their mobility in soils. For instance, the reduced redox potential of As (As^3+^ and As^5+^) has much higher permeability in soils than the increased redox potential of Cr (Cr^6+^ and Cr^3+^) (Wang et al., 2020). Furthermore, the oxidation state of soils can influence the redox potential of metals and metalloids. For instance, it has been revealed that biochar converts Cr^6+^ to the less mobile Cr^3+^ through consistently transferring electrons, which may have been connected with oxygen-containing active functional groups on that biochar surface (Dong et al., 2017). Furthermore, microbial metabolism utilizing biochar-derived organic carbon material can reduce Cr^6+^. The poor Cr solubility led to the reduction process, and Cr immobilization in soil has been enhanced. The adsorption and desorption mechanisms of metalloids and metals in soils are also significantly influenced by pH and organic matter because the adsorption of positively charged metals in biochar is high in acidic soil. In acidic soils (pH 3.5–6.0), Cr occurs predominantly in the positively charged forms Cr_3_(OH)_4_^5+^ and Cr(OH)^2+^ (Wang et al., 2020). The biochar amendment to soil could perhaps alter the dissolved organic content (DOC) and pH, thus resulting in the mobility of metals and metalloids.

According to some research findings, biochar-amended soil could improve the mobility of metals and metalloids such as Sb, As, and Cu (Beesley et al., 2013; Sun et al., 2018). For example, increased pH in biochar-amended soils led to increased As mobility (Beesley et al., 2013). Electrostatic interaction between anionic As and Sb elements and negatively charged biochar substrates may enhance effective desorption of As and Sb by increasing mobilization. In the case of Cu, mobility is strongly correlated with the DOC content in biochar. Cu can be immobilized by the adsorption process in the biochar (prepared at 600°C) surface with an elevated DOC content (Sun et al., 2018).

## Bio-/Phytoremediation With Biochar

Biochar aids in the bioremediation of organic and inorganic pollutants. The primary mechanism is an upsurge of microbial diversity that degrades hydrocarbons (petroleum) in biochar-amended polluted soils (Karppinen et al., 2017). Heavy metals and metalloids cannot be deteriorated or completely eradicated from the ecosystem, but they can be transformed from one form to another, from higher concentration to lower concentration. Heavy metals and metalloids can also accumulate in organisms (Verma et al., 2021). Hence, most frequently, two strategies are used for the heavy metal and metalloid bioremediation process (Li et al., 2019). Absorption and accumulation of metals and metalloids in timber plants and crops with bioenergy potential in metal-polluted farmlands, and their deduction by harvesting the biomass containing/accumulated with metals and metalloids, and the transformation of toxic metals and metalloids into lesser toxic products (complex form), which can be adsorbed by native microorganisms and further reduce their toxicity and migration (Sun et al., 2018).

**FIGURE 2:**
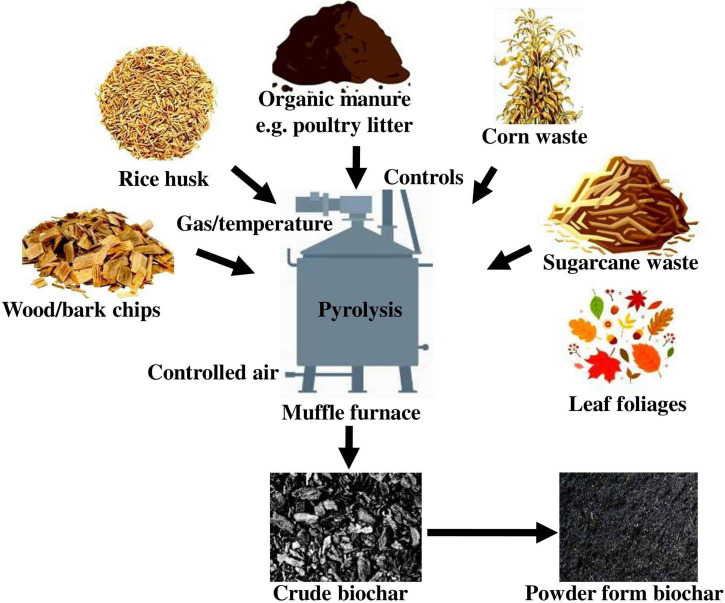
Possible benefits of biochar amendment in bio- and phytoremediation.

Cd^2+^ denotes cationic metal ion (A) physiological adsorption of cationic metals and metalloids of water from soil pores; (B) biochar co-precipitation with chloride, carbonates, silicate, and phosphate with metals; (C) complex formation with biochar surface functional groups; and (D) gradual nutritional discharge of DOC, N, Ca, P, and K for growth of plants and microbes in the root region (Figure 2). The mechanisms (A), (B), and (C) can minimize the bioavailable metal content in pore water, lowering phytotoxicity even further.

The negatively charged outer layer of biochar and its alkaline character can adsorb and sustain toxic metals through various mechanisms. Biochar, *via* gradually discharging nutrients and maintaining healthy soil structures and properties, also generates much more favorable soil conditions for the growth of beneficial microbes and plants (Das et al., 2021).

The existence of biochar significantly increased the lowering precipitation of Cr^6+^ to Cr^3+^ in the contaminated soils due to remarkably improved microbial activities encouraged by releasing carbon and other nutrients from biochar (Choppala et al., 2012). Furthermore, a decrease in the concentration may aid in the immobilization of metals and metalloids, including Cr^6+^ and U^6+^, but no evidence to date has demonstrated the role of biochar in bioremediation/phytoremediation. In addition to the effectively improved bioremediation, the existence of biochar does provide an indirect mechanism for metal and metalloid bioremediation (Sun et al., 2018; Gong et al., 2019).

Calcite precipitation caused by microbes can firmly adsorb and co-precipitate metals and metalloids on the surfaces. The metal ions along with an ionic radius similar to that of Ca^2+^, including Cu^2+^, Cd^2+^, and Pb^2+^, may be integrated into calcite crystal particles through alternative reactions during calcite precipitation (Achal et al., 2011). Biochar aided this strategy by making microbe-friendly soil conditions and potentially increasing bioremediation efficiency (Arif et al., 2020). *Bambusa vulgaris* biochar with an O_2_-releasing bead has been recently demonstrated as a promising O_2_-releasing substance used in soils and groundwater bioremediation (Wu et al., 2015). This kind of biochar does have the potential to enhance the oxidation level (from As^3+^ to a less mobile form) of metals and metalloids.

### Influence of Biochar in Bio-/Phytoremediation

**FIGURE 3:**
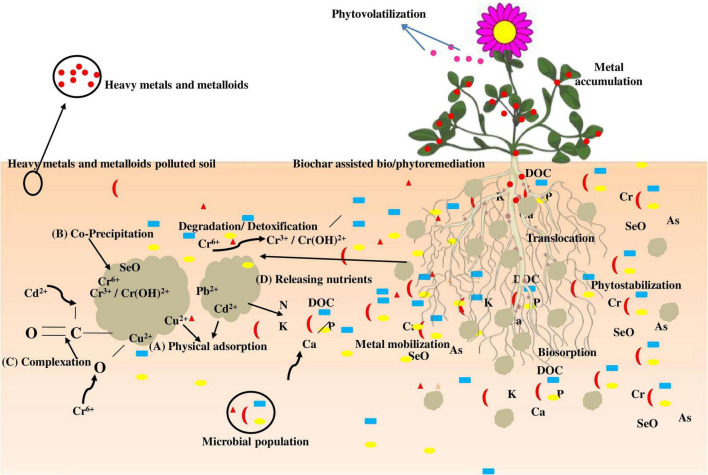
Phytoremediation potential of hyperaccumulator on metal-contaminated soil with and without the amendment of biochar.

A few research studies have investigated on biochar-augmented phytostabilization of metals and metalloids (e.g., Zn, As, Ni, Cd, Sb, Cu, and Cr) in contaminated soils (Uchimiya et al., 2012). Figure 3 represents the possible influence of biochar on bioremediation/phytoremediation of metal-contaminated soil. Arsenic (As) is well-recognized to react differently from some other metals and metalloids since the mobility of As can be diminished in acidic soils, owing to the enhanced sorption process on ferric oxide under an acidic environment. Hartley et al. (2009) demonstrated that biochar can also be applied for phytostabilization with *Miscanthus* species. Moreover, the analysis revealed that adding biochar derived from hardwood to soil samples did not improve As transport to *Miscanthus* plants, whereas alkaline biochar can mobilize As in metal-contaminated soils (Hartley et al., 2009). Cu and Pb were relatively straightforward to be stabilized in biochar-administered soils, whereas Cd and Ni differed widely depending on the nature of biochar used (Uchimiya et al., 2012; Sun et al., 2018). The stabilization mechanism is often probably due to increases in soil pH. The detailed research has shown that soil alterations (addition of lime) can be merged with phytoremediators to considerably reduce the bioavailability of metals and metalloids. Furthermore, biochar is also more effective at governing the accessibility of toxic compounds, as well as enhancing plant biomass fabrication and restoration performance (Břendová et al., 2015; Maddalwar et al., 2021).

Plant yield increases with biochar supplementation (Ambika et al., 2022) are connected to water and nutrient retainment, enhanced biological activities, and neutralized soil pH. Hence, biochar has the efficiency to be used as an amendment to reduce metal bioaccumulation in plants. Moreover, alterations in soil pH in the rhizosphere can feasibly influence the metal and metalloid mobilization efficiency of biochar in soils, while rhizosphere acidification should be avoided (Houben and Sonnet, 2015). Biochar is thought to interact with soils and balance their properties for an extended period of time. Thus, the redox mechanisms may cause biochar to change, a process called aging (Gul et al., 2015). The immobilization of heavy metals and metalloids in biochar has been associated with the lability of metals (e.g., Pb^2+^ is more mobile than Cd^2+^). A wide range of functional groups, including hydroxyl, carboxylic, and phenolic groups could be established during the aging process, and biochar aging had no effect on the immobilization of positively charged metals and metalloids in soils containing aged biochar (Heitkötter and Marschner, 2015; Fan et al., 2018).

### Biochar-Assisted Phytoremediation

Phytoremediation is a multidisciplinary field with the goal of mobilizing and/or immobilizing pollutants from different environmental conditions (Shah and Daverey, 2020). Phytoremediation encompasses phytostabilization, rhizoremediation, phytoextraction, phytodegradation, and phytovolatilization in general (Shah and Daverey, 2020). In comparison to certain other remediation practices for heavy metals and metalloids, including chemical immobilization, digging, and dumping, phytoremediation is gaining popularity due to its efficiency and lower cost (Wu et al., 2015). Other advantages, including erosion control and pollutant leaching prevention, are critical for future soil management and development. Table 2 summarizes some biochar-assisted phytoremediation plants for metal- and metalloid-polluted soils (Sun et al., 2018).

**TABLE 2:** Biochar-assisted phytoremediation for metal- and metalloid-polluted soils.

Name of plant	Phytoremediation on metal- and metalloid-contaminated soil	Biochar and dose	Effects on phytoremediation	References
Anthyllis vulneraria, Noccaea rotundifolium and Poa alpina	Ni, Cd, Ti, Zn, Cr, Pb, Cu, and Fe	Pruning residues and manure: 1.5–3%	Reduced water-extractable Zn, Cu, Cd, and Cr. Increased pH	Fellet et al., 2014
*Lolium perenne* L. var. Cadix	Pb and Cu	Oka, Ash, and Birch: 20% v/v	Reduced pore water-mediated Pb and Cu doses in shoots	Sun et al., 2018
*Solanum lycopersicum*	As, Cd, Zn, and Cu	Hardwood	Raised pore water with Cu and As. Immobilize Zn and Cd owing to elevated DOC and pH	Beesley and Marmiroli, 2011
*Oryza sativa*	As, Zn, Cd, Ni, Cr, Co, Pb, and Cu	Sewage sludge: 5 and 10%	Reduced pore water Pb, As, Ni, Cr, and Co owing to elevated soil pH. Mobilize Cd, Cu, and Zn	Khan et al., 2013
*Brassica juncea*	Cd, Pb, and Cu	Poultry manure and green waste	Increased (353%) plant shoot dry biomass. Decreased Pb, Cd, and Cu accumulation in plants	Park et al., 2011
*Brassica napus*	Cd, Zn, and Pb	Miscanthus: 5 and 10%	Reduced metals bioavailability in shoot biomass	Bandara et al., 2017
*Miscanthus*× *giganteus*	As	Hardwoods: 20%	Improved pore water with As	Sun et al., 2018
*Lycopersicon esculentum*	Cr, Mn, and Ni	Wood: 2.5–5%	Reduce exchangeable Cr, Ni, and Mn. Enhanced plant growth	Bandara et al., 2017

### Biochar-Assisted Phytoextraction

The primary method for remediating soil contamination is the phytoextraction process, which is typically associated with the ability of hyperaccumulators and energy plants to bioaccumulate metals and metalloids (Rezania et al., 2016). Numerous plant species were also used to extract various metals (e.g., Cr, Cd, Pb, As, Co, Cu, Zn, and Ni) from soils (Cameselle and Pena, 2016). Plant species preferably being used for phytoextraction should not just accumulate significant concentrations of the target metals and metalloids; nevertheless, they also have an increased biomass yield, tolerate the toxic effects of metals and metalloids, should be adaptable to soil and climatic conditions, are resistant to insects and pathogens, and will also be suitable for cultivation (Ranieri et al., 2020). The effectiveness of phytoextraction is determined by two factors: yield and metal and metalloid concentrations (Cameselle and Pena, 2016). Thus, the uptake of metals and metalloids, which is the outcome of the two factors, can sometimes be positive or negative (Coumar et al., 2016). Based on this, we identify that neither research findings fulfill all of the aforementioned criteria. Nevertheless, one study found that, while biochar-amended metal-polluted soil enhanced the willow plant biomass, the concentrations of Cd and Zn in willow were constant. Nonetheless, phytoextraction is improved (Břendová et al., 2015). In practice, phytoextraction is frequently used in farmland soils to reduce hazardous metal and metalloid concentrations below soil quality standards, thereby improving soil environmental quality and ensuring food security (Sun et al., 2018).

Phytoextraction of heavy metal-polluted soils, including mine sludge, could take centuries. Hence, the pollutant limits of the target agricultural fields should be kept to a minimum for phytoextraction (Mani and Kumar, 2014). However, a hyperaccumulator has the potential to acquire elevated metal and metalloid concentrations, but its slow growth rate frequently limits its application (Ranieri et al., 2020). Energy and economical plants, including sunflower and rapeseed, are often used to retrieve Cd from farmland soils. Recently, biochar-assisted phytoextraction has been emerging rapidly and used in practice. Accordingly, biochar-assisted *Brassica napus* was used to retrieve Cd metal-polluted agricultural soil (Houben and Sonnet, 2015). Various plant species and biochar are often used in multi-metal-contaminated soils. Nevertheless, limited research has focused on the combined effect of biochar on phytoextraction of heavy metal-contaminated soils (Houben and Sonnet, 2015; Sun et al., 2018). Correspondingly, *Amaranthus tricolor* was subjected to biochar-assisted phytoextraction to treat Cd-polluted agricultural soils (Lu et al., 2015). So far, many research findings showed that adding biochar to plants considerably reduces heavy metal and metalloid bioavailability. Nevertheless, some plants necessitate elevated doses of bioavailable metals and metalloids to accumulate them. The advantages of biochar include improved contaminated soil physicochemical properties, increased microbial population and activities, and increased ability to enhance agriculture production (Lu et al., 2015; Ye et al., 2016). Hence, using biochar to remediate metal- and metalloid-polluted soils not only immobilizes them but also increases microbial population, lowering the environmental threat of heavy metals and metalloids in soils even further (Frankel et al., 2016).

### Biochar-Assisted Phytostabilization

Phytostabilization is another phytoremediation method that is widely used for the stabilization of metals and metalloids in mine sludges (Barbosa and Fernando, 2018). The revegetation approach reduces dispersion and erosion because plant roots stop leaching, which contributes significantly to the immobilization of metals and metalloids (Sarkar and Sadhukhan, 2022). Precipitation, complexation, metal electron reduction, and root adsorption are the potential phytostabilization mechanisms (Ma et al., 2016). Phytostabilization, as opposed to phytoextraction, is more concerned with metal and metalloid sequestration in the rhizosphere than in other plant tissues (Yan et al., 2020). Metals and metalloids are typically stabilized by applying soil amendments (such as biochar and compost) and microbes *in situ*, which improve metal immobilization and plant growth (Figure 3; Kumpiene et al., 2019).

## Application of Biochar Aided Phytoremediation of Mine Sites

Mining (e.g., coal, gold, copper, magnesite, bauxite, and iron mining) activities can degrade soil quality and structure and disturb biological systems and vegetation, thus leading to widespread soil pollution (Gabarrón et al., 2019). Heavy metal toxicity and elevated acidity of soil contaminated by mining activity reduce the revegetation possibilities of metal-polluted soils. Remediation of such metal-polluted soils can be accomplished through phytoremediation, a long-term and cost-effective rehabilitation approach that promotes revegetation to minimize the chances of contaminant transfer and land reclamation. However, these are difficult to accomplish in the absence of appropriate soil amendments (e.g., biochar) (Fellet et al., 2014). The biochar amalgamation with heavy metal-polluted soil may improve pH fertility and water-holding capacity, minimize the mobility of pollutants, and encourage revegetation (Kelly et al., 2014). Phytoremediation of mine sludge soil with biochar obtained from residues of orchard prune and organic manure at four distinct concentration levels (0, 1, 5, and 10%) demonstrated substantial benefits of biochar in revegetating plant species in metal- and metalloid-contaminated soils. Also, the bioavailability of Zn, Cd, and Pb reduced proportionally as the biochar content increased (Fellet et al., 2014).

## Conclusion

One of the most important remedial technologies for heavy metal- and metalloid-polluted soils is biochar-blended bioremediation/phytoremediation. Biochar-stimulated phytore- mediation has a significant potential for immobilizing cationic heavy metals and metalloids in mine sludge soils and other metal-contaminated soils, especially those under high acidic conditions. Biochar can significantly decrease the bioavailability and leachability of cationic metals and metalloids in soils; enhance soil structure, physicochemical properties, fertility, and revegetation; and foster soil microbial populations. Nevertheless, since biochar appears to become less efficient in stabilizing highly harmful cationic metals and metalloids, which provide their mobility in soils, the implementation of biochar-aided phytoremediation is competent in attempting to resolve multi-metal-polluted soils. Furthermore, it is essential to select suitable biochar in order to develop a successful strategy for immobilizing anionic metals and metalloids initially through an *in vitro* approach. Moreover, more extensive research is required to assess the efficacy of biochar-amended bioremediation/phytoremediation of heavy metal-polluted soils. Scientific investigations should concentrate on the following important themes: (A) demonstrating the interrelations between raw materials used in pyrolysis, biochar physicochemical properties, and soil bioremediation/phytoremediation; (B) assessing the biochar consistency and its impacts on the transfer of metals and metalloids in mine sludge and metal-polluted soils in a field-level study; (C) knowing the mechanisms of biochar-influenced bioremediation/phytoremediation, particularly the interactions between biochar, microbial populations, plant roots, and soil particles.

## Author Contributions

Both authors listed have made a substantial, direct, and intellectual contribution to the work, and approved it for publication.

## Conflict of Interest

The authors declare that the research was conducted in the absence of any commercial or financial relationships that could be construed as a potential conflict of interest.

## Publisher’s Note

All claims expressed in this article are solely those of the authors and do not necessarily represent those of their affiliated organizations, or those of the publisher, the editors and the reviewers. Any product that may be evaluated in this article, or claim that may be made by its manufacturer, is not guaranteed or endorsed by the publisher.
